# Ageritin from Pioppino Mushroom: The Prototype of Ribotoxin-Like Proteins, a Novel Family of Specific Ribonucleases in Edible Mushrooms

**DOI:** 10.3390/toxins13040263

**Published:** 2021-04-07

**Authors:** Sara Ragucci, Nicola Landi, Rosita Russo, Mariangela Valletta, Paolo Vincenzo Pedone, Angela Chambery, Antimo Di Maro

**Affiliations:** Department of Environmental, Biological and Pharmaceutical Sciences and Technologies (DiSTABiF), University of Campania ‘Luigi Vanvitelli’, Via Vivaldi 43, 81100-Caserta, Italy; sara.ragucci@unicampania.it (S.R.); nicola.landi@unicampania.it (N.L.); rosita.russo@unicampania.it (R.R.); mariangela.valletta@unicampania.it (M.V.); paolovincenzo.pedone@unicampania.it (P.V.P.); angela.chambery@unicampania.it (A.C.)

**Keywords:** *Agrocybe aegerita*, *Cyclocybe aegerita*, antiviral activity, antifungal activity, endonuclease activity, cytotoxicity, protein structure function

## Abstract

Ageritin is a specific ribonuclease, extracted from the edible mushroom *Cyclocybe aegerita* (synonym *Agrocybe aegerita*), which cleaves a single phosphodiester bond located within the universally conserved alpha-sarcin loop (SRL) of 23–28S rRNAs. This cleavage leads to the inhibition of protein biosynthesis, followed by cellular death through apoptosis. The structural and enzymatic properties show that Ageritin is the prototype of a novel specific ribonucleases family named ‘ribotoxin-like proteins’, recently found in fruiting bodies of other edible basidiomycetes mushrooms (e.g., Ostreatin from *Pleurotus ostreatus*, Edulitins from *Boletus edulis*, and Gambositin from *Calocybe gambosa*). Although the putative role of this toxin, present in high amount in fruiting body (>2.5 mg per 100 g) of *C. aegerita,* is unknown, its antifungal and insecticidal actions strongly support a role in defense mechanisms. Thus, in this review, we focus on structural, biological, antipathogenic, and enzymatic characteristics of this ribotoxin-like protein. We also highlight its biological relevance and potential biotechnological applications in agriculture as a bio-pesticide and in biomedicine as a therapeutic and diagnostic agent.

## 1. Introduction

Since always, nature inspires medicine for the diversity of biologically active compounds with therapeutic properties and the great attention that researchers paid on mushrooms is one of the most appropriate examples, which deserves to be further investigated. Many fungal species are traditionally used in Chinese medicine or as functional foods in Japan and other Asian countries, representing a great source of nutraceuticals, functional foods, and secondary metabolites. In this context, fungi are useful to discovering new drugs; and many bioactive compounds such as small molecules, polysaccharides, proteins, and polysaccharide–protein complexes have been isolated from them [1,2]. A number of bioactive proteins and peptides, including lectins, fungal immunomodulatory proteins (FIPs), ribosome-inactivating proteins (RIPs), antifungal and antimicrobial proteins, laccases, ribonucleases, and ribotoxins have been isolated and characterized from mushrooms [3]. Among them, fungal ribotoxins are a family of highly specific extracellular rRNA endonucleases (EC 3.1.27.10) well studied since 1960. They are produced by fungi belonging to Ascomycota phylum, mostly from the genus *Aspergillus*, such as the prototype α-sarcin from *Aspergillus giganteus* [4] and restrictocin and mitogillin from *Aspergillus restrictus* [5], while hirsutellin A and anisoplin are isolated by *Hirsutella thompsonii* and *Metarhizium anisopliae*, respectively [6,7]. Specifically, ribotoxins exert their toxicity by entering the cells and cleaving a single phosphodiester bond between G_4325_ and A_4326_ (rat 28S rRNA numbering) located in the Sarcin Ricin Loop (SRL) on the larger RNA subunit (rRNA) of the ribosome [8]. This region is necessary for EF-G or EF-2 elongation factors’ ribosome interaction during mRNA–tRNA translocation in prokaryotes and eukaryotes, respectively [9]. The cleavage leads to the release of a 460-nt fragment, known as α-fragment, at the 3′ end of the 28S RNA, causing the inhibition of protein biosynthesis, followed by cellular death through an apoptotic pathway [10,11]. From a structural point of view, ribotoxins are basic proteins of ~150 amino acids residues with a high degree of identity and an ordered secondary structure including two conserved disulphide bridges with a β-sheet, where is located the active site, and a short α-helix [12]. On the other hand, the biological function of ribotoxins is not yet clear, although several studies highlight their defence role as insecticidal and antifungal agents [12,13]. Considering that, until now, no protein receptors exist for ribotoxins, the alteration of cell permeability produced, for example, by tumour transformation or a viral infection facilitates these toxins to cross membranes, making them anticancer or antiviral agents [12].

Recently, a novel family of protein synthesis inhibitors has been discovered in fungi belonging to Basidiomycota phylum. These enzymes, the prototype of which is Ageritin from *Cyclocybe aegerita* (synonym *Agrocybe aegerita*), are specific ribonucleases, named as ribotoxin-like proteins (RL-Ps), that, as ascomycetes ribotoxins, basically act on large rRNA, releasing the specific α-fragment, although the two families differ in structure and mechanism of action (see Section 5 and Section 6) [14,15].

## 2. The Edible Mushroom *Cyclocybe aegerita*

*C. aegerita* (V. Brig.) Singer 1951 (synonym *A. aegerita*), commonly known as Pioppino in Italy, is a popular edible mushroom belonging to the Basidiomycota division of fungi (order Agaricales) [16]. This mushroom is well known and commonly found in Southern Europe, South-Eastern USA, as well as in similar climatic zones of Asia, preferring warm or mild temperate climates [17]. In nature, this mushroom is a saprotrophic or facultative pathogenic fungus causing a white-rot of hardwood of deciduous trees, especially in *Populus* and *Salix* spp. in forests with warm and mild climate.

The basidiocarp is characterized by a hemispherical or convex–flat cap, at first brown to lighten when ripe. The stem is a fibrillose and solid, white or very pale brown with a broad and membranous ring, initially white and then brown for the spores. The gills are white when young and brown or dark brown when ripe, while the spores are ellipsoidal, smooth, and show tobacco brown [18,19].

Known as an excellent edible mushroom due to its taste and fragrance, Pioppino mushroom is at the same time a reservoir of several bioactive metabolites such as indole derivatives with free radical-scavenging [20,21] and polysaccharides [22]. Due to the presence of secondary metabolites, several beneficial qualities are attributed to *C. aegerita*, such as anti-inflammatory, antifungal, antibiotic, and anti-tumor properties [23]. Moreover, a study by Landi and coworkers indicates that wild Pioppino from Campania region is a reservoir of many nutrients such as amino acids, malic acid, and sugars with free radical scavenging properties [24]. In addition, Pioppino mushroom is also a source of many enzymes (e.g., peroxidases, laccases, proteases, and lipoxygenase) with possible biotechnological applications [25], proteins/enzymes with a specific activity in inhibiting tumor growth [26,27], and an excellent experimental system. Indeed, *C. aegerita* is an archetypal mushroom well studied as model for investigating mushroom developmental biology [28].

Finally, the taxonomic classification of the fungal species is still unclear due to the lack of resolute data. Novel evidences proposed the name *C. aegerita* (V. Brig.) Vizzini for indicating this species [18]. Nowadays, these assignments were recently updated by some authors [29,30,31] and future comparative genomics analyses will be performed to get definitive information and assign the correct taxonomy and the name of this species. In particular, the reclassification of Pioppino mushroom has begun on the basis of DNA analysis reported by Vizzini and Angelini [18] that transferred this edible mushroom from the genus *Agrocybe* to the *Cyclocybe* one, establishing the scientific name *Cyclocybe aegerita*.

## 3. Isolation of Ageritin from *Cyclocybe aegerita* (V. Brig.) Singer

At the beginning of 2017, Landi and co-workers reported the isolation of a novel toxin, named Ageritin, from the edible mushroom *C. aegerita* [14]. This is the first documented evidence of the presence in the Basidiomycota phylum of a specific ribonuclease that following ribosomes incubation causes the release of the diagnostic α-fragment, inhibiting protein synthesis in vitro [14]. These activities are similar to those exerted by α-sarcin [32], the prototype of ribotoxins family from Ascomycetes filamentous fungi [12]. Due to its peculiar structural and functional characteristics, Ageritin differs from Ascomycetes ribotoxins, named by us ‘classic ribotoxins’, and is considered the prototype of a novel family of specific ribonucleases, called ‘ribotoxin-like proteins’ isolated from basidiomycetes [14]. Ageritin can be purified at homogeneity from *C. aegerita* fruiting bodies by using a well-established protocol for the purification of basic proteins [33]. In particular, total proteins extracted in a phosphate-saline buffer were precipitated at pH 4.0 using acetic acid. The obtained soluble proteins were fractionated by cation exchange chromatography on Streamline SP and eluted with 1M NaCl. Subsequently, a gel-filtration chromatography on Sephacryl S-100-HR allowed isolating a pool of active proteins with an elution volume of ~17 k. Subsequently, the pooled fractions were dialyzed and subjected to cation exchange chromatography on an AKTA purifier system. Purified Ageritin was obtained with an estimated amount of about 1.3 mg/100 g of fresh fruiting bodies. Protein synthesis inhibition was monitored by applying the procedure reported previously [14].

In 2019, Ragucci and co-workers optimized the purification procedure of Ageritin by employing a scale-up protocol optimized to shorten purification times and to increase the protein amounts [34]. For this purpose, a gel-filtration step using a Superdex75 Hiload 26/60 column on an AKTA purifier system, followed by a cation exchange chromatography step on a SP-Sepharose fast flow, allowed a significant increase in the protein purification yield (2.5 mg/100 g of fresh fruiting bodies) [34]. By this procedure, we also purify at homogeneity an Ageritin isoform, after named Met-Ageritin, with an additional *N*-terminal methionyl residue, as unique structural difference with respect to Ageritin primary structure. However, this apparently unique inequality dramatically alters the enzymatic features of Met-Ageritin despite, as Ageritin releases the α-fragment and inhibits protein synthesis in vitro with an IC_50_ of 2.8 nM, a value 21-fold higher than that of Ageritin (see Section 5.1) [35].

## 4. Cellular Localization of Ageritin

**Figure 1:**
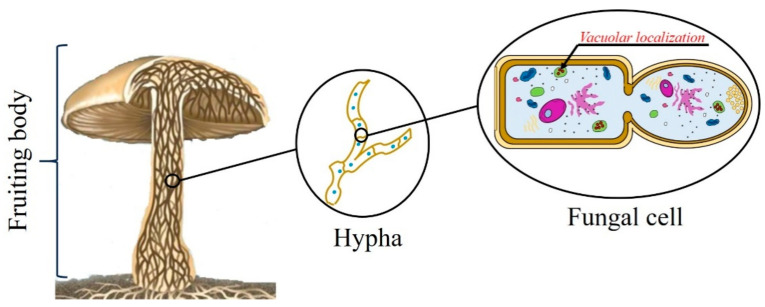
Schematic representation of Ageritin intracellular localization in hyphal fruiting bodies of *Cyclocybe aegerita*. Ageritin (in red) is mainly localized in vacuoles of fungal cells.

Since it is widely accepted that Ageritin is able to act on ribosomes of both eukaryotes and prokaryotes (see Section 6.1) and to investigate its potential toxicity against *C. aegerita* ribosomes, Baglivo and co-workers studied Ageritin cellular localization. Interestingly, the protein was found inside *C. aegerita* hyphae [36]. More in detail, positive signals were mainly localized in the cellular vacuole by immunolocalization studies (see the graphical scheme in Figure 1). These findings can explain the lack of the toxic activity against *C. aegerita* ribosomes. Furthermore, the extra 21 amino acids *N*-terminal peptide, not found in the purified protein (see Section 5.4), could be a signal peptide for vacuolar delivery in edible mushrooms [36]. As an alternative, the same peptide could be implicated in unconventional transport/secretion processes [37,38,39], although no known *consensus* sequences for subcellular localization were found by bioinformatics approaches [39].

## 5. Structural Properties of Ageritin

### 5.1. Physicochemical Characteristics of Ageritin

The physicochemical properties of Ageritin are summarized in Table 1. This enzyme, like other classic ribotoxins, is a non-glycosylated, basic, and monomeric protein of 135 amino acids residues with an experimental molecular weight of 14,801.8 Da ([M+H]^+^) [40] and an isoelectric point ≥9.5 [14]. Ageritin inhibits protein synthesis in a rabbit reticulocyte lysate system with an IC_50_ of 133 pM, a value higher than other classic ribotoxins from ascomycetes (range 10–40 pM) [14,41]. This toxin specifically hydrolyzes a single phosphodiester bond of the large subunit of ribosomal RNA (rRNA) in the SRL, releasing the diagnostic α-fragment, similarly to classic ribotoxins from ascomycetes, whose prototype is α-sarcin. Moreover, thermal and chemical denaturation studies by circular dichroism measurements certified the high thermal and chemical stability of Ageritin. Notably, the high thermal stability with an elevated Tm (~78 °C) at pH 7.4 is peculiar among ribotoxins, characterized by Tm values ranging from 52 to 62 °C [40,42]. On the other hand, the protein possesses very high resistance against the action of denaturant as revealed by the high Cm value of 5.5 M, at pH 7.4 [40]. These Cm values are higher than Cm values of small stable enzymes such as RNase A and lysozyme (3.0 M and 4.2 M, respectively [43]).

## 5.2. Metal Dependence

The high isoelectric point of classic ascomycetes ribotoxins suggests that their interactions with the ribosome likely depend on strong electrostatic nature [44]. Accordingly, these enzymes are typically inactivated by cations, which impair their activity by competing with the electrostatic interaction between ribotoxin and ribosomes [8,11]. Unexpectedly, Ruggiero et al. observed that, in contrast to classic ribotoxins, the activity of Ageritin is completely suppressed by EDTA, according to Ageritin capability to bind metal ions [40]. In particular, the isothermal titration calorimetry binding studies revealed the presence of binding sites for Ca^2+^, Mg^2+^, and Zn^2+^ in protein structure (Table 2). These data support the metal dependence of Ageritin, able to bind cationic ions, with the strongest affinity for Zn^2+^ [45]. In particular, zinc ions regulate diverse mechanisms of fungal pathogenesis in which zinc-binding proteins are typically involved [46]. As a zinc-binding virulence factor, Ageritin activity is impaired by chelating agents, like EDTA, and is strongly enhanced at low zinc concentrations. Moreover, at high zinc concentrations, non-specific effects are observed, likely due to the partial inhibition of the interaction between the enzyme and SRL. The requirement of metal binding for the ribonucleolytic activity of Ageritin was further ascertained using a synthetic 35-mer SRL analog, used as a suitable substrate to investigate the effects of metal ions on catalytic activity [45]. On the other hand, the inactivation by chelating agent (EDTA) of heterologous protein obtained by Tayyrov et al. was not retrieved [47]. This could be justified by the presence of the extra N-terminal peptide of 21 amino acid residues likely able to produce configuration and conformational changes in this different protein.

### 5.3. Protein Structure, 3D Homology Modelling and Phylogenetic Analysis

The main structural features of Ageritin are summarized in Table 3. Differently from other classic ribotoxins and homologous fungal RNases T1 family, which contain two highly conserved disulphide bridges, the analysis of Ageritin amino acid composition reveals that the protein has 135 amino acid residues with a single free reactive cysteinyl residue [40,48]. Moreover, the toxin possesses a ratio Lys+Arg/Asp+Glu of 1.4 with an expected prevalence of basic amino acid residues and an extinction coefficient (ε^0.1%^) of 1.47 L g^−1^ cm^−1^, experimentally obtained by amino acid analyses [39]. The calculated negative grand average of hydropathicity (GRAVY) index of −0.26 suggests an overall hydrophilic nature for this protein.

**Figure 2:**
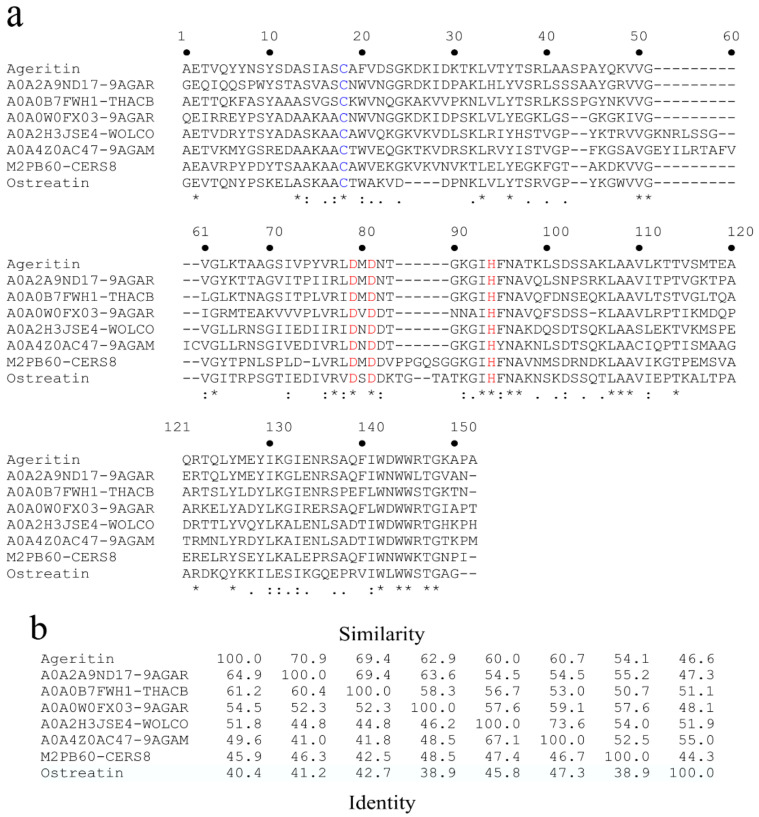
(**a**) Sequence alignment of native Ageritin with similar proteins retrieved by BLAST similarity searches. The standard one-letter code was used for the amino acid residues; identical residues (*), conserved substitutions (:), and semi-conserved substitutions (.) are reported. Single free cysteinyl residues are highlighted in blue; the conserved catalytic triad is highlighted in red. A0A2A9ND17_9AGAR (*Amanita thiersii*), A0A0B7FWH1_THACB (*Thanatephorus cucumeris*), A0A0W0FX03_9AGAR (*Moniliophthora roreri*), M2PB60_CERS8 (*Ceriporiopsis subvermispora*), A0A4Z0AC47_9AGAM (*Hericium alpestre*) and A0A2H3JSE4_WOLCO (*Wolfiporia cocos*) are uncharacterized proteins from several basidiomycetes group. Ostreatin RL-P from *Pleurotus ostreatus* [15]. (**b**) Identity/similarity matrix for the comparison of Ageritin with similar proteins retrieved by BLAST similarity searches. The identity/similarity matrix of seven uncharacterized proteins from Basidiomycota species is shown. Identity and similarity values are reported below or above the diagonal axis, respectively, representing the percentage of identical or similar amino acid residues, respectively.

With the aim to determine Ageritin amino acid sequence, a combined approach based on the screening of the *C. aegerita* genome [16] and mass spectrometry was used [49,50]. In particular, by aligning the *N*-terminal sequence of native Ageritin with predicted amino acid sequences of the seven most homologous proteins from other basidiomycetes (Figure 2A), identity from ~65 to 40% and similarity from ~70 to 45% (Figure 2B) were observed [14].

No substantial similarity/identity of Ageritin sequence with other classical ribotoxins was observed, although it is well characterized the highly specific RNase enzymatic activity (see Section 6.1).

**Figure 3:**
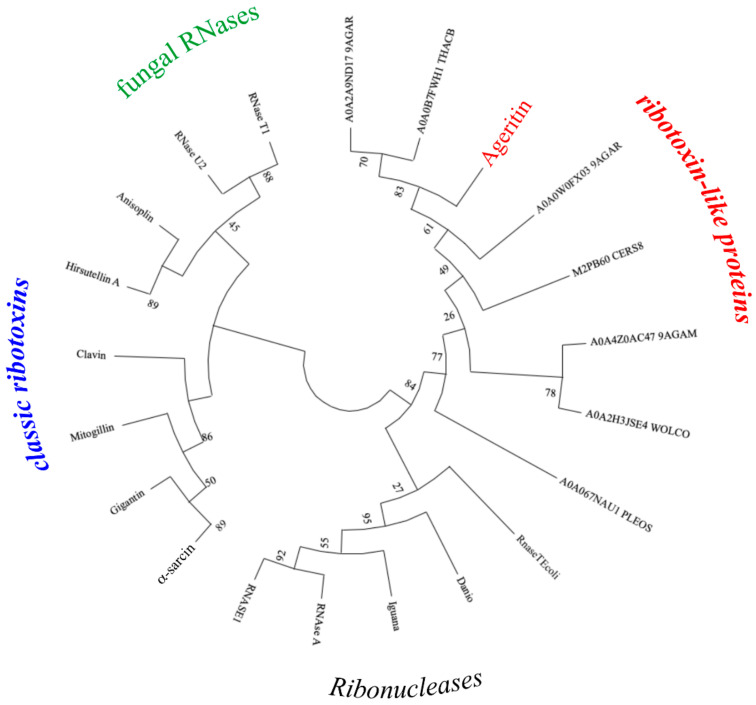
Rooted circular phylogenetic tree of RNases superfamily. The tree shows the close relationship of some RNases with classic ribotoxins, ribonucleases, and RL-P Ageritin (highlighted in red) together with other similar proteins retrieved in database. RL-Ps appear to be more distant with other analysed endonucleases, while RNases and fungal ribonucleases are more closely related to classic ribotoxins. The Maximum Likelihood method was used with Poisson-corrected distances. A0A2A9ND17_9AGAR (*Amanita thiersii*), A0A0B7FWH1_THACB (*Thanatephorus cucumeris*), A0A0W0FX03_9AGAR (*Moniliophthora roreri*), M2PB60_CERS8 (*Ceriporiopsis subvermispora*), A0A4Z0AC47_9AGAM (*Hericium alpestre*) and A0A2H3JSE4_WOLCO (*Wolfiporia cocos*) are uncharacterized proteins from several basidiomycete groups. Ostreatin RL-P from *Pleurotus ostreatus* [15]. α-sarcin, gigantin, mitogillin, clavin, hirsutellin A, and anisoplin are ribotoxins from several ascomycete groups, accession number P00655, P87063, P67876, P0CL70, P78696 and E9FCV0, respectively. RNase U2 and RNase T1 fungal RNases from *Ustilago sphaerogena* (AC: P00654) and *Aspergillus oryzae* (AC: P00651), respectively. Danio (RNS L3; AC: A5HAK0; *Danio rerio*, Zebrafish), Iguana (RNAS1; AC: P80287; *Iguana iguana*, Common iguana) and RNAse A (AC: P61823; *Bos taurus*, bovin) as well as RNAS1 (AC: P07998; *H. sapiens,* human) are vertebrate RNases. RnaseTEcoli (AC: P30014; *Escherichia coli*) is a bacterial RNase.

Phylogenetic relationships analysis of 20 sequences of proteins belonging to ribonucleases superfamily revealed that four main groups (i.e., ribonucleases, fungal RNases, classic ribotoxins and RL-Ps) could be distinguished (Figure 3). A common ancestor between Ageritin and related proteins was retrieved and these proteins are not closely related to other members of this clade, probably due to a convergent evolution process.

**Figure 4:**
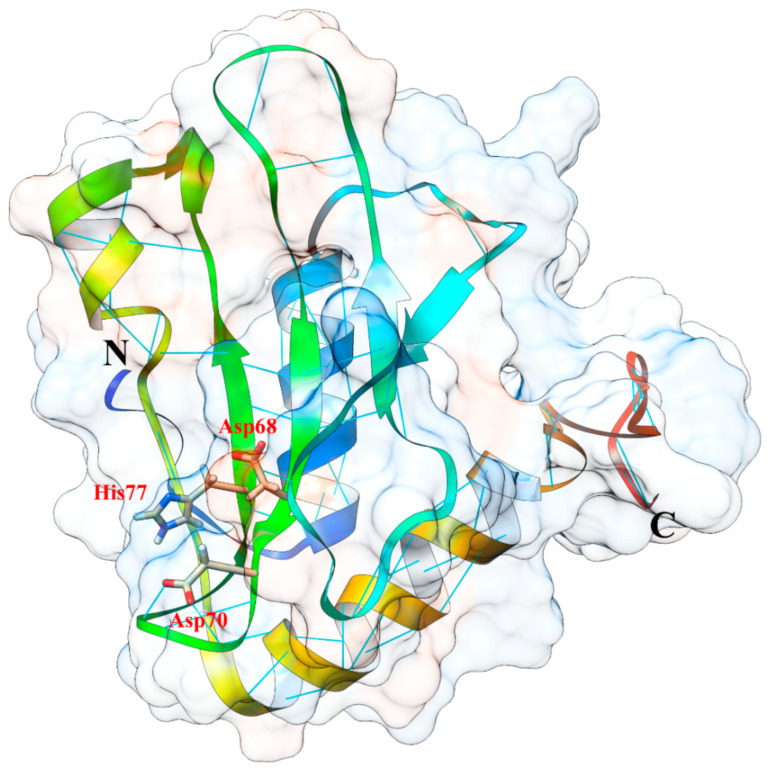
Semi-transparent surface overlaying a cartoon representation of Ageritin 3D-molecular model. The catalytic triad residues (one histidinyl and two aspartyl residues) are labeled and represented as sticks.

The unavailability of 3D-structure in protein database and the difficulties during extensive studies of X-ray crystallography (difficulty in obtaining the crystal) led to perform the 3D Ageritin structure by homology modeling (Figure 4). The approach revealed that Ageritin has an α/β fold with a structural core made of an antiparallel β-sheet interacting with an adjacent long α-helix, as ascomycetes ribotoxins and fungal RNases. However, for Ageritin the β-sheet is orthogonal with respect to this α-helix, unlike the other classic ribotoxins [45,49]. Deeper structural analyses by Landi et al. and Ruggiero et al. revealed that the catalytic residues located in a pocket of the β-sheet are Asp68, Asp70, and His77, forming the catalytic triad (Figure 4) [45,49] as also confirmed by mutagenesis studies of Tayyrov et al. [47].

Moreover, the predicted Ageritin 3D structure shares the highest similar fold with the contact-dependent growth inhibition A (CdiA) toxin from *Escherichia coli*, with the predicted catalytic residues also conserved [45,49].

### 5.4. Gene Organization

Sequence analysis by Baglivo and co-workers revealed that the gene encoding for Ageritin consists of four exons (43,133, 39 and 255 bp) and three short introns (60, 55 and 69 bp) placed at the 5′ region of the coding sequence and conserved splice junctions in agree with other fungal sequences [36]. This sequence codes for a precursor of 156 amino acids (~17-kDa) containing an extra 21 amino acid peptide at the *N*-terminus of the protein, not present in the purified polypeptide (135 amino acid residues; ~15-kDa) as confirmed by mass spectrometry and Western blot analysis using anti-Ageritin rabbit polyclonal antibodies [36].

## 6. Enzymatic Properties of Ageritin

### 6.1. Ribonucleolytic Activity of Ageritin on Ribosomes

The ribonucleolytic activity of ribotoxins is extremely specific, acting on the highly conserved SRL located in the larger rRNA of all ribosomes. Specifically, these enzymes cleave a single phosphodiester bond of the GAGA tetraloop, causing the release of an approximately 400 nucleotide-long fragment from the 3′ end of rRNA (depending on the ribosome origin), known as α-fragment [12]. The cleavage of this phosphodiester bond inhibits protein biosynthesis and induces cell death by apoptosis [51]. Similarly to classic ribotoxins, Ageritin is able to recognize the GAGA tetraloop and to cleave the single phosphodiester bond conserved in ribosomes belonging to all kind of organisms. In particular, Ageritin was tested on: (i) ribosomes from rabbit reticulocyte lysate [35]; (ii) isolated fungal ribosomes from yeast *Saccharomyces cerevisiae* [14] and *Trichoderma asperellum* [35]; and (iii) isolated bacterial ribosomes from *Escherichia coli* and *Micrococcus lysodeikticus* [52]. In all cases, the α-fragment detected on polyacrylamide gel in denaturing conditions confirms the specificity of the ribonuclease action.

### 6.2. Ribonuclease Activity on Tobacco Mosaic Virus (TMV) RNA

Mycoviruses (fungal virus) are the viruses that specifically infect fungi and many of them have RNA as their genetic material [53]. Using the positive strand RNA of Tobacco Mosaic Virus (TMV) as a model, Citores et al. demonstrated that Ageritin is able to promote an extensive degradation of the polyphosphate RNA backbone and therefore to increase its mobility [52]. Moreover, corroborating the metal dependence of Ageritin (see Section 5.2), magnesium ions are activators of Ageritin antiviral activity, whose effect is reversed by the chelating agent EDTA. A possible explanation of this activity is that the target of ribotoxins, the GNRA tetraloop motif is common in all kinds of three-dimensional RNA structures [54]. The activity on the viral RNA displayed by Ageritin, suggests a role in fungal defense towards viruses.

### 6.3. Endonuclease Activity on Plasmid and Genomic DNAs

Examples of proteins involved in host defense against foreign nucleic acid molecules can be retrieved in literature. For instance, Lee et al. describe a protein from *Helicobacter pylori* with ribonuclease and nicking endonuclease activities on RNA or plasmid DNA, respectively [55]. In addition, Hsia et al. report some sugar-nonspecific nucleases in prokaryotes able to cleave both RNA and DNA. Moreover, it is worth mentioning that, sharing with ribotoxins the SRL loop as target, some RIPs, besides their ribonuclease activity on rRNA, display nicking endonuclease activity on supercoiled plasmid DNA [56,57,58]. Accordingly, Citores et al. demonstrated that the magnesium-dependent nicking endonuclease activity of Ageritin on plasmid pCR2.1 was also reverted by EDTA [52]. In another work, Ragucci et al. confirmed that Ageritin enzymatic activity on pUC18 plasmid was susceptible to the presence of both Zn^2+^ and Mg^2+^ ions, with a more evident effect for the latter [34]. Other antifungal proteins were also reported to bind DNA causing cell death by promoting nucleic acid charge neutralization and subsequent condensation [59]. Similarly, Ageritin is active on *E. coli*, *P. digitatum* and COLO 320 cells genomic DNAs. The observed high genomic DNA degradation activity was strongly magnesium dependent and, as expected, was reverted by EDTA [52].

## 7. Antiproliferative and Defense Activities of Ageritin

### 7.1. Cytotoxic Activity

Many studies documented the cytotoxicity of Ageritin toward several tumour cell lines as summarized in Table 4. For instance, Landi et al. [14] describe the effects of Ageritin toward different neural and glial tumour cell lines, while Citores et al. [52] tested the effects of Ageritin on COLO 320, HeLa and Raji cells. Moreover, a novel property of Ageritin has emerged in a recent work by Ragucci et al. investigating the cytotoxic action of Ageritin against SH-SY5Y neuroblastoma cells (undifferentiated or retinoic acid differentiated) showing a selective cell toxicity against undifferentiated cells [34]. The selective toxicity of Ageritin versus malignant cells was also confirmed by Lampitella and co-workers that described the significant effects of the toxin on cancer SVT2 cells viability, while no or slight effects were observed on normal BALB/c 3T3 cells [39]. In the same study, immunofluorescence experiments proved the selective internalization of Ageritin into cancer cells, in association with its selective cytotoxic activity. Therefore, this selective action against tumour cells could make this toxin a novel potential tool for anticancer drugs. Overall, these studies suggest that apoptosis acts as the main mechanism responsible for cell death induced by Ageritin. Cytotoxicity results varied among tested cell lines, with HeLa cells being the most sensitive cells and IC_50_ ranging from nanomolar to micromolar (Table 4). In a pivotal study, Landi and co-workers also highlight the pro-apoptotic effect of Ageritin towards central nervous system (CNS) model cell lines mediated by the activation of caspase-8 (extrinsic pathway) and by the induction of nuclear fragmentation [14].

The activation of apoptosis pathway induced by Ageritin was further confirmed by the caspase-3 enzymatic activity and protein levels of cleaved PARP in SH-SY5Y cells [34]. Moreover, the irreversible pancaspase inhibitor Z-VAD-FMK was able to prevent the apoptotic pathway activation induced by Ageritin, confirming that the process was completely caspase-dependent [34].

In addition, Citores et al. hypothesized that besides apoptosis, cell death induced by Ageritin was also mediated by necroptosis [52]. Indeed, Ageritin-treated HeLa cells exposed phosphatidylserine on the cell surface, as revealed by an increase in the level of annexin VFITC-positive cells, suggesting the involvement of apoptosis. In addition, the visualization of propidium iodide (PI) staining indicated late stage apoptosis or necrosis. The involvement of caspase-dependent apoptosis was confirmed by the high caspase-3/7 activity in both COLO 320 and HeLa cells, with the cytotoxicity prevented by Z-VAD. However, following the addition of the necroptosis inhibitor Necrostatin (Nec-1), there is a strong decrease of cell death mediated by Ageritin [52]. Then, Citores et al. tested Ageritin toxicity on HeLa cells following the addition of some substances interfering with intracellular routing, such as the fungal inhibitor Brefeldin A and the ionophore monensin in order to study the Ageritin intracellular pathway [52]. In particular, Brefeldin A was shown to cause Golgi complex disassembly associated with an increase in protein cytotoxicity, indicating that Ageritin follows a Golgi-dependent pathway to the cytosol. It was reported that monensin possesses a pH-neutralizing effect in endosomes/lysosomes at high concentrations, lacking this effect at low concentrations, although it influences Golgi structure and function [60]. According to previous findings on the importance of Golgi transport for translocation, it was observed that low concentrations of monensin-pretreated HeLa cells enhanced the cytotoxicity of Ageritin. On the contrary, high ionophore concentrations sensitized the cells to Ageritin, indicating that the protein does not require a low pH for translocation to the cytosol [52]. A study by Lampitella and co-workers [39] suggests that Ageritin is also able to enter malignant cells by altering cell membranes, as occurred in model membranes treated with α-sarcin [61,62], bovine seminal ribonuclease [63,64] and chimeric constructs consisting of an amino-terminal type 1 RIP domain fused to a *C*-terminal protease inhibitor domain [65]. Indeed, studying the changes induced by Ageritin, in thermotropic phase transition of liposomes as models of normal and cancer eukaryotic cell membranes, Lampitella and co-workers demonstrated that Ageritin is unable to interact with normal eukaryotic model membranes (DPPC/Chol liposomes). On the other hand, there is a strong perturbation of cancer cell liposomes (DPPC/DPPS/Chol) due to the preferential interaction of basic Ageritin with anionic DPPS, which mediate protein translocation to the cytosol [39]. It was also reported that Ageritin interacts with bacterial model membranes, albeit only superficially, supporting the finding that Ageritin is not cytotoxic against several bacterial cells (see Section 7.4) [39,52].

### 7.2. Antifungal Activity

The antifungal activity of Ageritin (see Table 5) represents one of its most interesting features, since it is currently believed that fungal ribonucleases such as ribotoxins have no direct effects on fungi. Nevertheless, in 2018, Citores et al. reported that α-sarcin was endowed with antifungal activity towards the green mold *P. digitatum*, entering the cytosol and inactivating the ribosomes, and killing the cells by arresting the fungal growth [13].

As described for α-sarcin, Ageritin inhibited the growth of *P. digitatum* by acting on major rRNA and irreversibly inactivating fungal ribosomes, thus inhibiting protein synthesis. Indeed, Ageritin was less active than α-sarcin and a concentration 60-fold higher was required to inhibit fungal growth. This difference of activity could be possibly related to their diverse ability to cross cell membranes [52]. Subsequently, Ragucci et al. tested the antifungal activity of Ageritin against the filamentous fungus *Trichoderma asperellum* and the single-celled eukaryote fungus *Saccharomyces cerevisiae.* This study demonstrated that Ageritin is able to exert inhibitory action only against the *T. asperellum* although the protein concentration able to inhibit 50% of fungal growth was about 40-fold higher than that required for *P. digitatum* growth inhibition [34]. These findings are in agreement with the defense role hypothesized for Ageritin, also considering that during cultivation, edible mushrooms could be contaminated by several ascomycetes fungi, such as *Trichoderma* species, that cause fungal fruiting bodies damage by inhibiting its growth [66]. RNAs from Ageritin-treated *P. digitatum* were also isolated and analyzed confirming the release of α-fragment mediated by Ageritin in vivo [52].

### 7.3. Entomotoxic and Nematotoxic Activity

Entomotoxic activity is a common feature of classic ribotoxins from ascomycetes [67], such as anisoplin [7] and hirsutellin A [42], both toxic towards insect cells. In light of this, Tayyrov and coworkers recently demonstrated that, although less active than α-sarcin, Ageritin also exerts strong toxicity against *Aedes aegypti* larvae and *Spodoptera frugiperda* Sf21 cells [47]. These findings suggest a defense role for Ageritin in *C. aegerita* mushroom towards insect antagonists, like fungus gnats. On the other hand, since nematodes are important predators of fungi, Tayyrov and co-workers tested the nematotoxicity of Ageritin, considering that it structurally differs from other ribotoxins, which not display this activity [47,68]. However, as already reported for classic ribotoxins, Ageritin is inactive, indicating that nematode ribosomes are either resistant or not accessible to Ageritin [47].

### 7.4. Antibacterial Activity

Bacteria can cause significant yield losses in mushrooms crops, being responsible for a wide range of mushroom diseases [69]. Since bacterial ribosomes are sensitive to Ageritin (see Section 6.1), the antifungal activity of this ribonuclease (native form) was tested on several species such as *Micrococcus lysodeikticus*, *Escherichia coli*, *Pectobacterium carotovorum*, *Serratia marcescens*, and *Rhizobium leguminosarum* [52]. In another work, both native and alkylated Ageritin were tested on a panel of selected Gram-negative (*E. coli* ATCC 25922, *Salmonella enterica* 706 RIVM and *Pseudomonas aeruginosa* 01) and Gram-positive (*Bacillus globigii* TNO BMO13, *Staphylococcus aureus* ATCC 12,600 and MRSA WKZ-2) strains [39]. Among all tested bacteria, native Ageritin is toxic only against *M. lysodeikticus* with a concentration of 5.3 µM leading to 50% of growth inhibition. Moreover, a very limited susceptibility against the alkylated protein was only observed for the MRSA WKZ-2 strain, likely due to a different interaction of the ribotoxin with the bacterial plasma membrane and/or cell wall and its ability to cross them [39,52].

## 8. Other Members of Ribotoxin-Like Proteins from Edible Mushrooms

In order to confirm the expression of RL-Ps in basidiomycetes edible mushrooms, other members of this novel family of ribonucleases have been isolated and characterized. In particular, Landi and co-workers purified and characterized a RL-P, named Ostreatin, from *Pleurotus ostreatus* [15]. Similarly to Ageritin, Ostreatin is also a basic (pI value ≥ 9.0) and monomeric (~15-kDa) protein that releases the α-fragment from SRL following yeast or rabbit ribosomes incubation. This toxin also exhibits a low endonuclease activity on plasmid DNA both with and without the presence of metal ions (Mg^2+^ and Zn^2+^). Structurally, Ostreatin shares with Ageritin the same catalytic triad (D62, D64 and H75) [47,49] and a single reactive free cysteinyl residue (Cys18) [40]. Moreover, a comparison between the primary structure and the gene sequence encoding for Ostreatin revealed that, like Ageritin, also this toxin is synthesized as a pre-form, post-translationally processed by proteolysis. The additional *N*-terminal peptide of 16 amino acids shares a similarity with that of Ageritin [36]. Furthermore, Ostreatin 3D homology modeling reveals a similar fold compared to Ageritin, with a core consisting of an antiparallel β-sheet interacting with a parallel α-helix. At genome level, the nucleotide sequence of *ostreatin*-gene is characterized by 3 exons and 2 introns located at the 5′ region [36].

**Figure 5:**
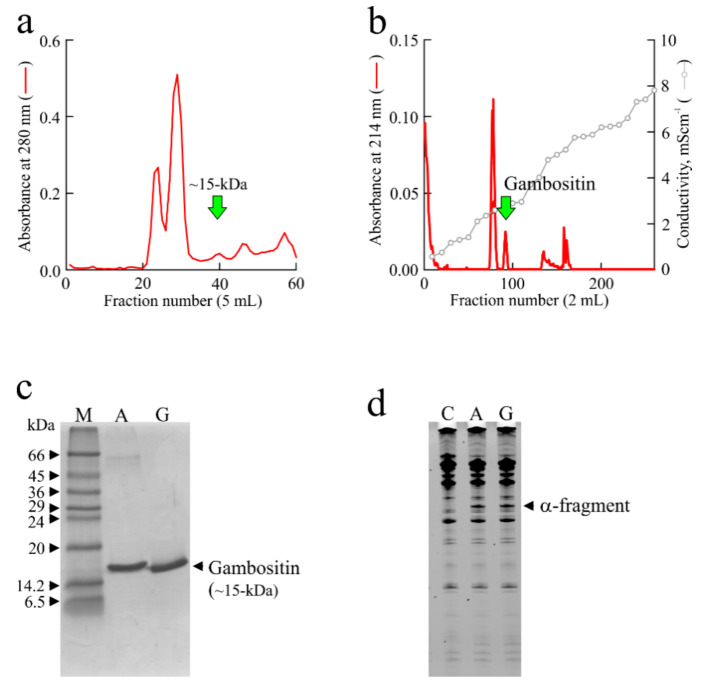
Purification of Gambositin from *Calocybe gambosa* fruiting bodies. Following the protocol described in Section 3, we reported: (**a**) elution profile of gel-filtration chromatography performed on HiLoad 16/60 Superdex 75 prep grade (GE Healthcare, Italy). The arrow highlights the peak containing proteins of about 15 kDa. (**b**), elution profile of the last purification step (exchange ion chromatography on S-Sepharose column; GE Healthcare). The arrow highlights the peak containing Gambositin RL-P that releases the α-fragment from ribosomes. (**c**) SDS–PAGE analysis in 15% polyacrylamide separating gel of either Ageritin (A; 3.0 μg) or Gambositin (G; 3.0 μg), under reducing conditions. M, molecular weight markers. (**d**) rRNA RL-P activity of Gambositin on rabbit ribosomes. Each lane contains 3.0 μg of RNA isolated from either untreated (control; C) or RL-P treated ribosomes. Ribosomes were treated with 2.0 μg of Ageritin (A) or Gambositin (G).

Further evidence of the presence of RL-Ps in edible mushrooms is provided by the isolation of two novel members of this family from *Boletus edulis* edible mushroom able to release the α-fragment, named Edulitin 1 and 2 with a molecular weight of ~16-kDa and ~14-kDa, respectively [70]. Finally, another member, named Gambositin (~15-kDa; few µg per 100 g of fruiting bodies), was isolated from *Calocybe gambosa* edible mushroom (Figure 5). *C. gambosa* fruiting bodies (known as ‘Virni’ in Italian) well known and highly appreciated in Campania region (Italy) for their taste and flavor.

## 9. Possible Applications of Ageritin in Medicine and Agriculture

Ageritin is a very stable and non-glycosylated protein with a single reactive cysteinyl residue, which possesses peculiar structural features useful for potential biotechnological applications.

This toxin could be used as part of immunotoxins (chimeric proteins obtained by linking a toxin to a carrier molecule) [71], or conjugates with other suitable carriers such as peptides, specific proteins or nanomaterials [72]. This is necessary given the toxicity of Ageritin alone towards various cell lines (see Table 4). Indeed, the utilization of immunotoxins/immunoconjugates would eliminate the harmful cells in a selective manner. In this framework, the antibodies or conjugates used should provide the specificity required to recognize unwanted cells with respect to normal cells.

Furthermore, considering the rising global food demand due to the population expansion, new control strategies must be tested for plant crops protection towards pathogens or pests, avoiding the use of environmentally unfriendly phytochemicals [12]. Therefore, a promising approach to pathogens or pests control could be the *ageritin*-gene transfection in plant cells, considering that the protein product possesses insecticidal activity [47]. This would be possible considering the recent progress in the areas of in vitro cultures and genetic plant engineering. A similar experimental approach is also useful for the controlling fungal diseases given the fungicide activity of Ageritin [52]. Last, but not least, the proposed applications should be accepted by the public opinion since ‘Ageritin’ toxin is found in Pioppino edible mushroom normally eaten. Overall, the general framework encourages further studies on Ageritin and homologous members belonging to RL-Ps family.

## 10. Concluding Remarks

In this review, we summarized the principal findings reported for Ageritin, the first member of a novel family of specific ribonucleases named ‘ribotoxin-like proteins’, isolated from basidiomycetes edible mushrooms. Since its isolation, the knowledge about its structure, enzymatic mechanism, and biological and antibiological action continues to increase.

On one hand, there is still an unresolved issue. Indeed, no biological role has yet been assigned to Ageritin, although its possible importance in giving an evolutionary advantage to the mushroom, would justify the conservation of RL-Ps family in these organisms. On the other hand, considering that several studies on enzymatic and biological activity of Ageritin have been done, we suggest that this protein/toxin has a function in the mushroom defense against predators, mold, and viruses, acting as a modulator of *C. aegerita* mushroom adaptation and defense against pathogens and competitors.

Nevertheless, Ageritin represents a novel biological tool useful both as therapeutic agent in biomedicine and as bio-pesticide in a sustainable agriculture or in general, as an essential model for translational research.

## Figures and Tables

**Table 1:** Physicochemical properties of Ageritin purified from *Cyclocybe aegerita* fruiting bodies.

	M*r* (Da)		pI		IC_50_ (pM)	Tm (°C)	Cm ([GuHCl]_1/2_/M)
	Exp	Theor	Exp	Theor		pH 7.4	pH 7.4
Ageritin	14,801.8	14,800.8	>9.5	9.3	133	78.1	5.5

M*r*: relative molecular mass; pI: isoelectric point; IC_50_: half-maximal inhibitory concentration; Tm: melting temperature; Cm: GuHCl concentration at half completion of unfolding transition. Exp: experimental; Theor: theoretical.

**Table 2:** Isothermal Titration Calorimetry (ITC) values of Ageritin in the presence of Mg^2+^, Ca^2+^, and Zn^2+^ ions.

ITC Experimental Values			
	N of Sites	K_ITC_ (M^−1^)	ΔH (Kcal mol^−1^)
Mg^2+^	3	3.25 × 10^6^	−1.6
Ca^2+^	1	7.68 × 10^5^	−13.1
Zn^2+^	4	1.77 × 10^7^	−9.9

N of sites: number of protein binding sites for metal ions; K_ITC_: binding constant or isothermal titration calorimetry association constant, ΔH: enthalpy related to K_ITC_.

**Table 3:** Principal characteristics of Ageritin primary structure considering its amino acid composition.

Amino Acid Residues	Cys	Lys + Arg	Extinction Coefficient	GRAVY *
		Asp + Glu	0.1%	
135	1 Free	1.4	1.47	−0.26

* grand average of hydropathicity (GRAVY) value was obtained by analyzing the amino acid composition of Ageritin with ProtParam tool—ExPASy.

**Table 4:** Cytotoxic activity of Ageritin against different cell lines.

Cell Line	Organism	Tissue	Morphology	Disease	Culture Properties	IC_50_ at 48 h		Ref.
						µg/mL	µM	
SH-SY5Y	Human	Bone marrow	Epithelial	Neuroblastoma	Mixed, adherent/suspension	77.30	5.15	[34]
SK-N-BE(2)-C	Human	Bone marrow	Neuroblast	Neuroblastoma	Mixed, adherent/suspension	8.41	0.56	[14]
C6	Rat	Brain	Fibroblast	Glioma	Adherent	4.58	0.30	[14]
U-251	Human	Brain	Pleomorphic/ astrocytoid	Glioblastoma	Adherent	9.46	0.63	[14]
HeLa	Human	Cervix	Epithelial	Carcinoma	Adherent	0.06	0.004	[52]
Colo 320	Human	Colon	rounded and refractile	Adenocarcinoma	Mixed, adherent/suspension	28.50	1.90	[52]
Raji	Human	Lymphoblast	Lymphoblast	Lymphoma	Suspension	15.00	1.00	[52]
SVT2 *	Mouse	Embryo	Fibroblast	-	Adherent	26.00	1.73	[39]
Balb/c 3T3 **	Mouse	Embryo	Fibroblast	-	Adherent	78.00	5.20	[39]
Sf21	Fall armyworm	Ovary	-	-	Suspension	n.r.	n.r.	[47]

* Simian-virus-40-transformed mouse fibroblasts. **, parental non-transformed BALB/c 3T3 cells. n.r., not reported.

**Table 5:** Antifungal activity of Ageritin against different fungal species.

Organism	Strain	Observation in Petri Dish	(°C) **	Medium	Growth Conditions	Toxin Final Concentration (µM)	Growth Inhibition (%)	Ref.
Filamentous fungus	*Penicillium* *digitatum* *	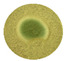	26	PDB ^a^; 150 µL	100 spores/well	2.9	79.0	[52]
						1.8	76.0	
						0.6	63.0	
						0.3	49.0	
Filamentous fungus	*Trichoderma* *asperellum* (TC74)	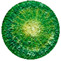	26	PDB ^a^; 150 µL	100 spores/well	13.3	46.6	[35]
						6.7	29.2	
						3.3	23.7	
Unicellular fungus	*Saccharomyces* *cerevisiae* (BY4741)	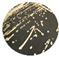	30	YPD ^b^; 5 mL	0.1 O. D. ^c^	13.3	~10	[35]

* Strain typified from Spanish Type Culture Collection (CECT), ** Optimal Growth Temperature; Valencia, Spain; ^a^ Potato dextrose broth; ^b^ Yeast extract peptone dextrose; ^c^ Initial optical absorbance at 600 nm.

## Data Availability

Not applicable.

## Abbreviations

Cm	GuHCl concentration at half-completion of the transition
RL-P	Ribotoxin-like protein
Tm	Melting temperature.
